# Uncovering Exposure Patterns of Metals, PFAS, Phthalates, and PAHs and Their Combined Effect on Liver Injury Markers

**DOI:** 10.3390/jox15060178

**Published:** 2025-11-01

**Authors:** Doreen Jehu-Appiah, Emmanuel Obeng-Gyasi

**Affiliations:** 1 Department of Built Environment, North Carolina A&T State University, Greensboro, NC 27411, USA; 2 Environmental Health and Disease Laboratory, North Carolina A&T State University, Greensboro, NC 27411, USA

**Keywords:** environmental mixtures, Bayesian Kernel machine regression, principal component pursuit, limits of detection, metals, PFAS, phthalates, liver disease

## Abstract

People are exposed to mixtures of metals, per- and polyfluoroalkyl substances (PFAS), phthalates, and polycyclic aromatic hydrocarbons (PAH) rather than single chemicals, yet mixture inference is hampered by high dimensionality, correlation, missingness, and left-censoring below limits of detection (LOD). We analyzed 2013–2014 National Health and Nutrition Examination Survey (NHANES) biomarkers (n = 4367) to (i) recover latent, interpretable co-exposure structures and (ii) quantify how these mixtures relate to liver health. To denoise and handle censoring, we applied Principal Component Pursuit with LOD adjustment (PCP-LOD), decomposing the exposure matrix into a non-negative low-rank component (population co-exposure profiles) and a sparse component (individual spikes), and then used Bayesian Kernel Machine Regression (BKMR) to estimate nonlinear and interactive associations with AST, ALT, GGT, ALP, total bilirubin, and the Fatty Liver Index (FLI), retaining analytes with ≥50% detection. PCP-LOD revealed coherent clusters (e.g., long-chain PFAS grouping; shared metal loadings), while the sparse layer highlighted episodic phthalate elevations. BKMR indicated outcome-specific mixture effects: PAHs and selected phthalates showed consistently positive associations with ALP, GGT, and FLI; PFAS (PFOS, PFNA, PFOA) exhibited modest associations with ALP and bilirubin; metals displayed mixed directions. A joint increase in the overall mixture from the 25th to 75th percentile corresponded to an upward shift in FLI and a smaller rise in ALT. This censoring-aware low-rank-plus-sparse framework coupled with flexible mixture modeling recovers actionable exposure architecture and reveals clinically relevant links to liver injury and steatosis, motivating longitudinal and mechanistic studies to strengthen causal interpretation.

## 1. Introduction

### 1.1. Characterizing Exposure Patterns: Methodological Challenges and Solutions

Human exposure to environmental chemicals is widespread and is often difficult to avoid. Rather than encountering isolated pollutants, individuals are typically exposed to diverse mixtures of metals, PFAS, phthalates, PAH, and other environmental pollutants through air, water, food, and consumer products across their lifespans [1,2]. These co-occurring exposures can interact biologically, resulting in cumulative, synergistic, or even antagonistic health effects that complicate traditional risk assessment frameworks [1]. Characterizing patterns within chemical mixtures is essential for identifying shared sources, tracing exposure pathways, and understanding how these complex interactions contribute to disease etiology. Environmental mixtures may increase hepatic disease risk by affecting biomarkers such as lipids, blood pressure, and cholesterol [2,3,4].

We analyze mixtures of metals, PFAS (per- and polyfluoroalkyl substances), phthalates, and PAHs (polycyclic aromatic hydrocarbons) using NHANES (National Health and Nutrition Examination Survey) data from CDC (Centers for Disease Control and Prevention); we handle <LOD (limit of detection) with PCP-LOD and model mixture effects with BKMR, including FLI (fatty liver index) and liver enzymes as outcomes.

Biomonitoring provides a non-invasive measure of internal chemical exposure through blood or urine analysis [5]. Large-scale initiatives such as NHANES have generated extensive biomonitoring datasets, particularly urinary biomarkers of metals, PFAS, phthalates, and PAH, which have become instrumental in population-level exposure and health assessments. Despite these advances, analyzing complex chemical mixtures remains methodologically challenging [6]. Environmental exposure data are typically high-dimensional and are often characterized by strong correlations among chemicals, missing data, and a substantial proportion of measurements falling below the laboratory’s LOD [7,8]. These censored values may introduce substantial uncertainty. Depending on the laboratory protocol, values < LOD may be omitted entirely or reported with greater uncertainty [9,10].

Traditional approaches to addressing <LOD data, such as single or multiple imputation, have limitations. A common practice involves substituting censored values with LOD/$\sqrt{2}$ [11]. While this method, was more accurate than LOD/2 for estimating central tendency and variance, and simpler than maximum likelihood approaches [12], it does not address the underlying structural complexities in the data. Traditional dimension reduction techniques, such as principal component analysis (PCA) and factor analysis (FA), have long been used to explore latent structures in exposure data, with PCA identifying linear combinations of variables that capture maximum variance and FA modeling observed variables as functions of latent factors. However, these approaches assume linear relationships, are sensitive to outliers, and perform poorly with censored values, incomplete data, or non-random missingness [7,13,14]. To overcome these limitations, more robust matrix decomposition methods have emerged, such as PCP, which decomposes an exposure matrix into a low-rank component capturing dominant co-exposure patterns and a sparse component isolating extreme or rare events [15]. Importantly, this approach does not assume Gaussian noise or full data availability, making it more adaptable to real-world environmental datasets. In parallel with structure learning using PCP-LOD, BKMR has been employed to model mixture effects in NHANES, highlighting nonlinearity, interactions, and variable importance through posterior inclusion probabilities (PIPs) [16].

Recently, PCP has been adapted for use in environmental health research through extensions that account for left-censored data, enforce non-negativity constraints for interpretability, and accommodate missingness, yielding what is now known as PCP-LOD [7]. This method recovers interpretable exposure patterns while isolating outliers and noise, which is essential for advancing mixture-based environmental epidemiology.

### 1.2. Role of Biomonitoring and Urinary Biomarkers

Biomonitoring is a valuable approach for assessing internal exposure to environmental chemicals, as it involves the direct measurement of chemical substances or their metabolites in biological samples. Among available matrices, urine sampling is particularly advantageous due to its non-invasive nature and repeatability. It is well-suited for evaluating short- to medium-term exposures, especially for chemicals with relatively short biological half-lives, such as phthalates and certain PFAS [17].

Large-scale population studies such as NHANES have made biomonitoring data broadly accessible. NHANES provides nationally representative measurements of urinary biomarkers, enabling robust evaluations of chemical exposure distributions and their associations with health outcomes across diverse demographic groups in the United States [18]. Specifically, urinary concentrations of metals, PFAS, phthalates, and PAH measured in the NHANES offer a valuable source for exploring co-exposure patterns. These biomarkers also facilitate investigations into their potential links with liver health indicators, including aspartate aminotransferase (AST), alanine aminotransferase (ALT), gamma-glutamyl transferase (GGT), total bilirubin, and metabolic dysfunction-associated steatotic liver disease (MASLD).

### 1.3. Challenges in Analyzing Mixtures

While large-scale biomonitoring datasets provide critical opportunities to study environmental exposures, analyzing chemical mixtures presents several methodological challenges. Mixtures are typically high-dimensional and characterized by strong correlations among constituents, substantial missing data, and a considerable proportion of values below the analytical LOD [8]. These characteristics complicate the identification of underlying exposure patterns and increase the risk of biased or unstable results. Substitution methods commonly used to address values < LOD such as replacing nondetects with LOD/$\sqrt{2}$ can distort distributional properties, bias estimates of central tendency and variability, and obscure meaningful exposure patterns [9,12].

Moreover, standard approaches often fail to differentiate rare but potentially important exposure events from background variability, further limiting their utility in real-world environmental health applications [19,20]. These limitations have motivated the development of more robust statistical methods capable of accurately recovering low-dimensional structures from noisy, incomplete, and censored data. Such methods are essential for advancing mixture-based epidemiological research, where the goal is to uncover interpretable exposure profiles that can inform public health interventions and regulatory policies. In response to these challenges, newer approaches such as PCP and Bayesian methods have gained traction. PCP, an advanced matrix decomposition technique, improves upon PCA by separating low-rank structure from sparse anomalies, allowing for more accurate recovery of latent exposure patterns even in the presence of outliers or missing values [7,15]. Meanwhile, BKMR and other semi-parametric models offer flexible, non-linear frameworks to assess the joint, interactive, and potentially synergistic effects of correlated exposures on health outcomes [21].

### 1.4. Objectives

In this study, we apply PCP-LOD to urinary biomarker data from the 2013–2014 cycle of the NHANES, focusing on exposure to metals, PFAS, phthalates, and PAH. Our objectives are

1. To identify latent patterns of chemical co-exposure across multiple classes of environmental pollutants.
2. To examine the associations between these exposure patterns and key hepatic health biomarkers including AST, ALT, GGT, total bilirubin, and MASLD.

## 2. Materials and Methods

### 2.1. Data Source and Preprocessing

We utilized biomarker data from the 2013–2014 cycle of the NHANES, selecting chemical biomarkers corresponding to metals (e.g., lead, mercury, cadmium), PFAS, phthalates, and PAH for mixture pattern analysis. The survey provided measurements of urinary and serum biomarkers covering a broad range of exposures, including trace metals, per- and polyfluoroalkyl substances (PFAS), phthalates, and other plasticizers, all pollutants that previous studies have associated with adverse cardiovascular outcomes. Laboratory analyses of these chemicals were carried out using standardized protocols.

For urinary metals, concentrations were determined with inductively coupled plasma mass spectrometry (ICP-MS; ELAN^®^ DRC II, PerkinElmer SCIEX, Concord, ON, Canada) after a simple dilution step, enabling accurate quantification of multiple elemental isotopes. Serum PFAS levels were obtained using solid-phase extraction followed by high-performance liquid chromatography coupled with tandem mass spectrometry (HPLC-MS/MS; Agilent 1200, Agilent Technologies, Santa Clara, CA, USA; API 5500, AB Sciex, Concord, ON, Canada). To approximate overall exposure, isomer-specific measures of PFOA and PFOS were combined. For phthalates and related plasticizer metabolites, urinary concentrations were assessed with HPLC-ESI-MS/MS (Surveyor HPLC and TSQ Quantum Ultra, Thermo Fisher Scientific, San Jose, CA, USA). The procedure included enzymatic deconjugation and automated solid-phase extraction, while isotopically labeled internal standards (Cambridge Isotope Laboratories, Tewksbury, MA, USA) ensured assay reliability and accuracy. Only biomarkers with available and valid LOD information were retained for inclusion in the analysis. Table 1 lists the specific analytes included in our mixture analysis, grouped by chemical class based on established toxicological categories and NHANES metadata.

The NHANES protocol was approved by the National Center for Health Statistics Research Ethics Review Board, and written informed consent was obtained from all participants. Public-use NHANES data are de-identified and publicly available. This study involved secondary analysis of publicly available, de-identified NHANES data and posed no new safety hazards.

### 2.2. Quality Assurance and Control (QA/QC)

All NHANES 2013–2014 laboratory measurements were generated under CDC Division of Laboratory Sciences protocols and NCHS oversight in CLIA-certified laboratories. Sample collection and processing followed standardized MEC procedures (pre-screened collection materials; prompt separation; frozen storage; monitored shipment). Analytical runs for metals, PFAS, phthalate, and PAH biomarkers used multi-point calibration, reagent blanks, and low/medium/high QC pools; isotopically labeled internal standards and spike-recovery checks were applied for mass spectrometry methods. Precision was monitored via field/lab duplicates and periodic blind splits; laboratories participated in external proficiency testing. NCHS audited labs, reviewed control charts and quarterly QC reports, enforced Westgard multi-rules, and required corrective action before any re-analysis.

Before public release, NCHS performed automated/manual data vetting, outlier review, and method documentation. Each analyte includes a constant LOD and a detection flag; values < LOD are reported as LOD/√2 per NHANES guidance. Missingness was minimal. In secondary analysis, we harmonized units, screened biologically implausible values, and adhered to NHANES design guidance. Left-censoring was addressed primarily with PCP-LOD (censoring-aware, non-negativity-constrained decomposition) and checked in sensitivity analyses against standard NHANES LOD/√2 substitution and alternate detection thresholds. For BKMR, we documented kernel/hyperparameter choices, set random seeds, and version-controlled code to ensure reproducibility.

### 2.3. Implementation of Principal Component Pursuit with Limit of Detection (PCP-LOD)

PCP is a robust matrix decomposition technique that partitions an observed data matrix into two components: a low-rank matrix L, which captures the dominant shared variance structure among variables (e.g., underlying co-exposure patterns), and a sparse matrix S, which isolates rare, extreme, or individual-specific deviations [15]. This decomposition is particularly advantageous for environmental mixture data, which often exhibit complex, nonlinear relationships and are susceptible to outliers and measurement noise, characteristics that traditional Principal Component Analysis (PCA) fails to accommodate due to its reliance on linearity and sensitivity to perturbations [15,22].

In the context of environmental biomonitoring, such as the NHANES, a persistent analytical challenge arises from the frequent occurrence of left-censored observations due to values falling below the LOD. It is estimated that a significant proportion of urinary biomarker concentrations in NHANES data, particularly for metals, PFAS, phthalates, and other PAH, are censored, potentially obscuring true exposure distributions and confounding pattern discovery [23,24]. Standard multivariate techniques either impute values below LOD or exclude them entirely, risking substantial bias and misrepresentation of underlying exposure profiles [25].

To overcome these limitations, we adopted an enhanced variant known as PCP-LOD. This method extends the classical PCP framework by directly modeling left-censored data and incorporating structural constraints to improve interpretability and robustness in chemical mixture analysis. Specifically, PCP-LOD incorporates non-negativity constraints on the low-rank matrix (L) to reflect the inherently non-negative nature of chemical concentrations, introduces LOD-aware penalization terms to explicitly account for censoring at the observation level rather than relying on ad hoc imputation, and natively handles missing data within the optimization process, thereby eliminating the need for pre-processing imputation [7,26].

Formally, the model assumes the observed biomarker matrix X can be expressed as the sum

X = L + S(1)

where

- L represents the low-rank component, capturing systematic co-exposure patterns across the population.
- S represents the sparse component, capturing unique or extreme exposure events at the individual level.

PCP-LOD offers a principled approach for identifying mixture patterns in the presence of LOD-related censoring and missingness, while preserving rare, potentially important exposure events—challenges commonly encountered in environmental health datasets.

### 2.4. Cross-Validation Objective, Robust Loss Function, and Parameter Selection

To ensure accurate pattern recovery, it is essential to select appropriate values for the tuning parameters λ (controlling sparsity), μ (controlling low-rank structure), and the target rank r (dimensionality of the low-rank component), which directly influence the decomposition and interpretability of the resulting exposure patterns. To identify the optimal combination of parameters, we implemented a grid search across candidate values. For each parameter setting, the PCP-LOD model was fit by solving a constrained matrix decomposition problem that separates the observed data matrix X into a non-negative low-rank component L and a sparse component S, while appropriately handling missing values and censoring below the LOD. The optimization procedure enforces non-negativity on L, accommodates missing entries, and integrates LOD thresholds into the loss calculation to ensure robust recovery of latent exposure patterns. The quality of each solution was evaluated using the LOD-aware loss function that considers only values above the detection limit:

(2) $$L_{LOD}\left(X,\hat{X}\right)=\sum_{i,j}\left\{\begin{array}{c} (X_{ij}-\;{\hat{X}}_{ij}{)}^{2},\;\;\;\;\;\;\;\;\;\;\;\mathrm{if}\;X_{ij}>\;{LOD}_{ij}\;\;\\ 0,\;\;\;\;\;\;\;\;\;\;\;\;\;\;\;\;\;\;\;\mathrm{otherwise}\;\;\;\;\;\;\;\;\;\;\;\;\;\;\;\;\;\;\;\;\;\;\;\;\;\;\; \end{array}\right.$$

Let X ∈ $R^{nxp}$ be the observed data matrix (e.g., biomarker concentrations), $\hat{X}$ = L + S is the reconstructed matrix from the PCP-LOD model, and ${LOD}_{ij}$ is the corresponding limit of detection for each variable. This ensures that only values above the detection threshold contribute to the loss function. The parameter combination with the minimum $L_{LOD}$ is selected as optimal. The goal was to select parameters $\left(\lambda^{*},\mu^{*},r^{*}\right)$ that minimize this LOD-aware reconstruction error:

(3) $$\left(\lambda^{*},\mu^{*},r^{*}\right)=\arg\underset{\lambda ,\mu ,r}{\min}L_{LOD}\left(X,\;\;\;{\hat{X}}_{\lambda ,\mu ,r}\right)$$

For each combination, the model was fit and the LOD-aware loss computed. The parameter combination yielding the lowest loss was selected as optimal and used to fit the final model for downstream analysis.

All data analysis and preprocessing were implemented in R (version 4.2.3; R Foundation for Statistical Computing, Vienna, Austria) leveraging custom functions sourced from the PCP-LOD master package [7], including functions.R and pcpr.R. Final data alignment and model input preparation were verified to ensure consistency in matrix dimensions prior to model estimation.

### 2.5. Bayesian Kernel Machine Regression

To assess the joint effects of multiple pollutants on hepatic outcomes, BKMR was applied to the refined exposure profiles obtained from the PCP analysis. BKMR is well suited for modeling high-dimensional mixtures, as it flexibly captures non-linear exposure–response relationships and interactions among correlated pollutants.

Let $Y_{i}$ denote the hepatic biomarker of interest (e.g., AST or total bilirubin), $X_{i}=(X_{i1},X_{i2},\ldots ,X_{iM})$ represent the mixture of M environmental exposures for individual i, and $Z_{i}$ denote covariates such as age, sex, BMI, and smoking status. The BKMR model is specified as

(4) $$Y_{i}=h(X_{i})+Z_{i}\beta +\varepsilon_{i},$$

where $h(X_{i})$ is an unknown, non-parametric exposure–response function modeled using a Gaussian process prior with kernel $K(X_{i},X_{j})$. The Gaussian kernel is defined as

(5) $$K\left(X_{i},X_{j}\right)=\exp\left.\left[-\sum_{m=1}^{M}1\frac{(X_{im}-X_{jm}{)}^{2}}{\rho }\right.\right],$$

where $K(X_{i},X_{j})$ measures the similarity between exposure profiles of individuals i and j, and ρ is a tuning parameter controlling the smoothness of the kernel (larger values yield smoother functions).

Posterior inference was performed within a hierarchical Bayesian framework to estimate pollutant-specific effects and their interactions, while quantifying associated uncertainty. This approach allows for a flexible examination of mixture effects on hepatic outcomes without imposing strict parametric assumptions.

### 2.6. Assessment of Liver Function and Steatosis

To assess hepatic function and delineate potential cases of metabolic dysfunction–associated steatotic liver disease (MASLD), six biomarkers were evaluated: aspartate aminotransferase (AST), alanine aminotransferase (ALT), gamma-glutamyl transferase (GGT), alkaline phosphatase (ALP), total bilirubin, and the FLI. These parameters are recognized clinical markers that collectively reflect hepatocellular injury, cholestatic processes, and overall hepatic metabolic integrity [27,28,29].

AST and ALT are aminotransferases released into circulation during hepatocellular injury, with elevated levels suggesting liver inflammation or necrosis [27]. GGT is a sensitive marker of biliary dysfunction and oxidative stress, often elevated in alcohol-related or metabolic liver disease [28]. ALP and total bilirubin further inform hepatobiliary integrity and cholestasis, with clinical relevance in both obstructive and infiltrative hepatic disorders [29].

In addition to these enzymatic markers, we derived the FLI as a non-invasive surrogate of hepatic steatosis. FLI was computed using the original Bedogni formula (Equation (6)), which combines anthropometric and biochemical parameters:

(6) $$\mathrm{FLI}\;=\;\frac{\exp\left(A\right)}{1+exp(A)}\times 100,$$

where

A = 0.953 × log (TG) + 0.139 × BMI + 0.718 × log (GGT) + 0.053 × waist circumference 2212 15.745

This algorithm incorporates triglycerides (TG, mg/dL), body mass index (BMI, kg/m^2^), GGT (U/L), and waist circumference (cm), offering validated diagnostic performance in identifying hepatic steatosis with thresholds of FLI ≥ 60 indicating high probability of fatty liver, and FLI < 30 suggesting its absence [30,31].

We read the biomarker changes considering standard medical cut-points, not just *p*-values. ALT and AST mainly reflect injury to liver cells; small shifts in a population can sit below diagnostic thresholds but still suggest higher risk when considered with other measures [27,29]. GGT can rise with bile flow problems and oxidative stress, but it is not specific on its own [28]. For fat in the liver, we use established FLI cut-points—FLI ≥ 60 (high chance of fatty liver) and FLI < 30 (low chance)—to judge the size and meaning of any change [30,31]. We describe small changes as subclinical (early-stage, not a diagnosis) unless they cross recognized clinical thresholds.

## 3. Results

### 3.1. Descriptive Statistics of Exposure Variables and Hepatic Biomarkers

Table 1 summarizes urinary concentrations of selected environmental chemicals grouped into four major classes: 13 heavy metals (e.g., arsenic, mercury, lead, thallium), 9 PFAS (e.g., PFOA, PFNA), 11 phthalates and plasticizers (e.g., MEP, MEHHP, MBzP), and 6 PAH metabolites (e.g., 1-hydroxynaphthalene, 3-hydroxyfluorene). Metal concentrations are reported in micrograms per liter (µg/L), while PFAS, phthalates, and PAH levels are expressed in nanograms per milliliter (ng/mL) or nanograms per liter (ng/L), as applicable. For each analyte, the table provides the sample size (*n*), arithmetic mean, and standard deviation (SD). These descriptive statistics characterize the underlying exposure landscape and serve as the foundation for mixture-based modeling of liver-related health outcomes in subsequent analyses.

### 3.2. PCP-LOD Model Results

#### 3.2.1. Latent Structure of Chemical Exposures

**Figure 1:**
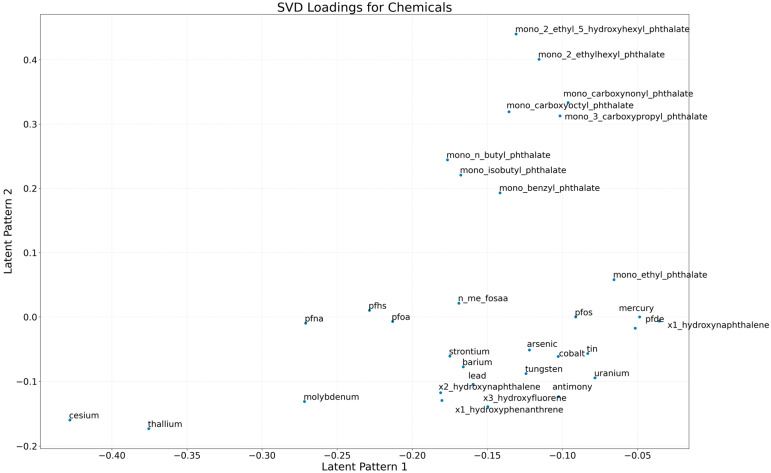
Singular value decomposition (SVD) loadings for biplot of chemical biomarker loadings on the first two latent dimensions, illustrating major co-exposure patterns and potential shared sources.

To identify dominant co-exposure patterns among chemical biomarkers, we applied Singular Value Decomposition (SVD) and examined the loadings of each chemical onto the first two latent dimensions [32,33]. This technique breaks the data into key components that capture the underlying structure of variation across all measured chemicals. The resulting biplot (Figure 1) shows how individual chemicals align with the first two dominant exposure patterns, or latent dimensions. Figure 1 reveals distinct clustering and separation of chemicals that reflect shared sources, exposure pathways, and physicochemical properties. The first latent dimension (x-axis) appears to contrast geogenic and industrial metals, such as cesium and thallium, which exhibit strong negative loadings, with synthetic PFAS like PFOA, PFDE, and PFUA, which load positively. Cesium and thallium, located on the far left, are known to derive from natural geological sources or contaminated food and water, and thallium is associated with coal combustion and smelter emissions [34,35]. In contrast, PFAS are industrial compounds widely used in firefighting foams, nonstick coatings, and consumer packaging, with human exposure primarily occurring via drinking water and dietary ingestion [36,37]. The second latent dimension (y-axis) differentiates bioaccumulative metals such as cadmium, antimony, and uranium, positioned in the upper half of the plot, from less persistent compounds, suggesting a gradient in retention or toxicokinetic [38,39]. Central clustering of elements like arsenic, tin, strontium, and barium points to mixed or overlapping exposure routes, including environmental degradation, industrial emissions, and contaminated soils [40]. Additionally, co-location of PFAS compounds (e.g., PFOA, PFHS, PFNA) supports evidence of concurrent use or exposure due to product formulation and environmental persistence, a pattern well-documented in biomonitoring studies [7]. SVD provided an interpretable latent structure that captures the principal axes of co-exposure variation in population-scale biomonitoring data.

#### 3.2.2. Detection Frequencies and Implications for Chemical Mixture Analysis

**Figure 2:**
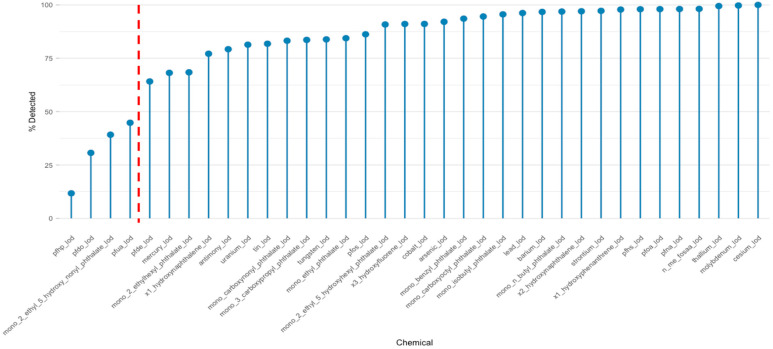
Detection frequencies of selected urinary biomarkers. A 50% detection threshold (red dashed line) was applied for downstream analyses.

Figure 2 displays the detection frequencies of selected urinary biomarkers from the 2013–2014 NHANES cohort, which illustrates the prevalence of a chemical in the population and helps determine which ones are reliable for analysis. A substantial number of chemicals—including several trace elements (e.g., cesium, molybdenum, thallium), phthalate metabolites (e.g., mono-benzyl phthalate, mono-isobutyl phthalate), and PFAS compounds—were detected in over 95% of participants. These high detection frequencies highlight widespread exposure across the U.S. population, likely stemming from persistent use in consumer products, contaminated food and water, and long biological half-lives that promote bioaccumulation [38,40,41].

In contrast, a subset of biomarkers, including perfluorohexanoic acid (PFHxA), perfluorobutanoic acid (PFBA), and mono-(2-ethyl-5-hydroxy-nonyl) phthalate, were detected in less than 30% of participants. Such low detection frequencies may indicate limited population-level exposure, rapid biological elimination, or challenges in analytical sensitivity for trace-level quantification. These findings align with known variability in detection across chemical classes and highlight the importance of properly handling LODs [12,42]. Left-censoring due to non-detects can introduce measurement bias, underestimate associations, and reduce the accuracy of latent structure estimation in mixture models [42,43]. To address this, we applied a ≥50% detection threshold as a criterion for inclusion in downstream analysis. This threshold balances statistical power with coverage of relevant environmental chemicals and aligns with prior studies employing PCP and BKMR to analyze complex mixtures [7,21]. The red dashed line in Figure 2 marks this 50% threshold, visually separating chemicals retained for modeling from those excluded. Chemicals found in most people give stable results in models, while those rarely detected may need special methods like imputation to avoid bias [19,41].

#### 3.2.3. Revealing Latent Correlation Structures Through Low-Rank Decomposition

**Figure 3:**
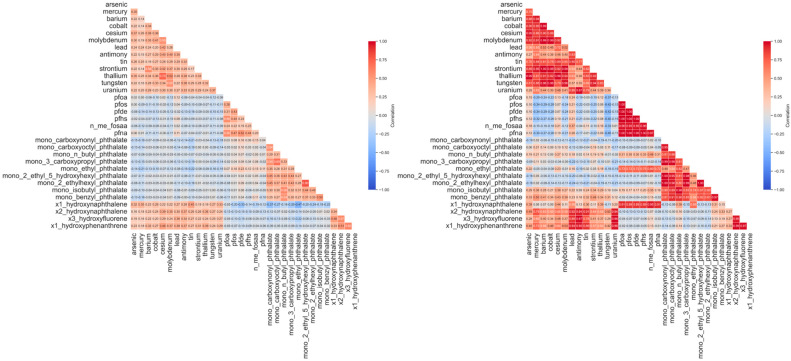
Left panel shows the raw Spearman correlation matrix; right panel shows the correlation structure after denoising.

**Figure 4:**
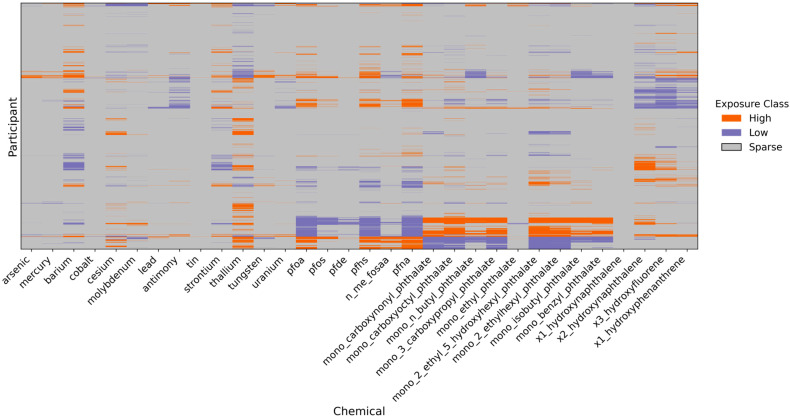
Classification of participant-specific exposure anomalies using the rescaled sparse matrix from PCP-LOD decomposition. Each cell represents a participant-chemical combination classified as High (orange), Low (purple), or Sparse (grey).

Figure 3 illustrates the pairwise Spearman correlation matrix for selected urinary biomarkers in the raw dataset. Overall, the raw correlations exhibit a diffuse structure, with most chemical pairs showing weak to moderate associations. A few moderate clusters emerge among heavy metals such as arsenic, lead, and mercury, and among certain PFAS and phthalate metabolites—suggesting possible shared environmental sources or common metabolic processing pathways [44,45]. However, the overall matrix is dense and noisy, reflecting the limitations inherent in biomonitoring datasets, including measurement variability, sporadic detection below LOD, and missing data [46]. Measurement noise and data sparsity can obscure true co-exposure patterns, making raw correlation estimates unstable and biologically difficult to interpret. To overcome this, we applied low-rank matrix decomposition to extract the dominant exposure structure. The resulting denoised correlation matrix (Figure 4) reveals clearer, more coherent chemical groupings by filtering noise and isolating the principal patterns of variation. For instance, heavy metals such as arsenic, lead, cadmium, and barium form a distinct high-correlation cluster, reflecting co-occurrence through shared industrial, dietary, or environmental exposure routes. Similarly, PFAS compounds—PFOA, PFNA, and PFHS—exhibit stronger internal correlations and are more clearly grouped than in the raw matrix. Importantly, many of the weaker, spurious correlations evident in the raw data are attenuated or removed, increasing signal-to-noise ratio and analytic clarity. The denoised correlation structure better reflects plausible biological and environmental co-exposures, aligns with known patterns in chemical biomonitoring literature [47,48].

#### 3.2.4. Characterization of Individual-Specific Chemical Anomalies via the Sparse Component

To identify extreme or individualized deviations in chemical exposures, we analyzed the sparse matrix Ŝ derived from the PCP-LOD decomposition. The sparse component represents person-specific deviations that are not explained by the low-rank structure and thus may capture rare or highly localized exposure events [7]. Figure 4 visualizes these events across participants and chemicals, revealing interpretable patterns of chemical-specific anomalies. We implemented an adaptive thresholding strategy informed by the distributional properties of the residual matrix:

(7) $$\mathrm{Residuals}=X_{scaled}-L$$

For each chemical j, we computed the standard deviation of the residuals (${SD}_{j}$) and rescaled the corresponding column in the sparse matrix S as

(8) $${\hat{S}}_{ij}^{rescaled}=\frac{S_{ij}}{{SD}_{j}}$$

This rescaling ensured that the thresholds used to define anomalous exposures were normalized relative to the expected variability of each chemical. Next, we defined high and low exposure events based on the empirical distribution of all rescaled sparse values across the matrix. Specifically, we used the 95th and 5th percentiles of the entire rescaled sparse matrix (i.e., across all participants and chemicals) as thresholds:

- Values above the 95th percentile were classified as High exposure events;
- Values below the 5th percentile were classified as Low exposure events;
- All other values were labeled as Sparse (representing the background or non-extreme cases).

(9) $${Class}_{ij}=\left\{\begin{array}{c} “High”,\;\;\;\;\;\;\;\;\;\;\mathrm{if}\;S_{ij}^{rescaled}>Q_{0.95}\;\;\;\\ “Low”,\;\;\;\;\;\;\;\;\;\;\mathrm{if}\;S_{ij}^{rescaled}<Q_{0.05}\;\\ “Sparse”,\;\;\;\;\;\;\;\;\;\;\;\;\;\;\;\;\;\;\;\;\;\;\;\;\;\;\;\mathrm{Otherwise}\; \end{array}\right.$$

The sparse matrix derived from PCP-LOD enabled precise identification of participant-specific anomalies in chemical concentrations, highlighting infrequent yet potentially important exposure events. High deviations were most evident for phthalates, indicating individualized exposure spikes. In contrast, PFOS, PFHS, and PFNA showed uniformly low or sparse signals across participants, suggesting population-wide exposure at consistently low levels. Chemicals such as cobalt, tin, and 1-hydroxynaphthalene were classified as sparse, reflecting low detection frequencies and minimal widespread exposure. For certain metals, including mercury, antimony, and uranium, the sparse matrix revealed low values with few high outliers, indicating background exposures with limited variability between individuals. To improve interpretability and account for left-censoring common in biomonitoring data, we implemented a three-tier classification framework. Values below the laboratory LOD were labeled Sparse, detectable values below the cohort median as Low, and those above the median as High. This approach distinguishes non-detects from quantifiable low-level exposures, addressing a critical challenge in mixture analysis where detection varies across compounds [38,43]. It aligns with best practices for interpreting human biomonitoring data, particularly when assessing heterogeneous exposure distributions in environmental mixtures.

#### 3.2.5. Component Loadings Represent Dominant Chemical Co-Exposure Patterns

**Figure 5:**
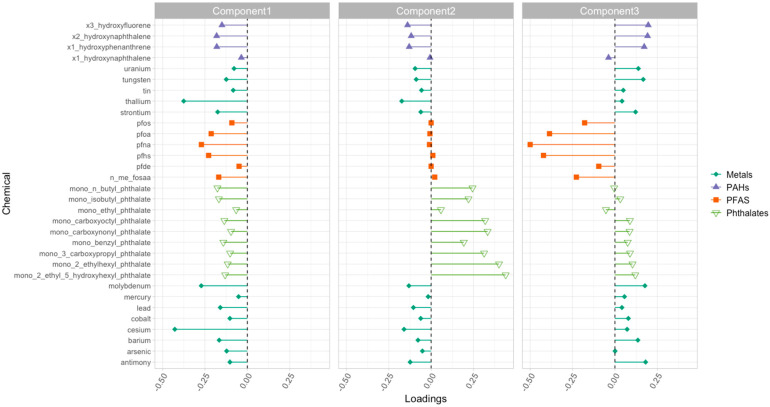
Principal components of the denoised matrix showing distinct co-exposure groupings. Each bar represents a chemical’s loading on the latent component. Component 1 shows a metals-dominated pattern—especially thallium and cesium—with generally negative loadings across classes. Component 2 is driven by phthalate metabolites (mostly positive loadings), while several metals tend to load negatively. Component 3 captures a PFAS pattern with mainly negative PFAS loadings and small positive loadings for some PAHs/metals.

Figure 5 shows the loadings of three principal components derived from the chemical concentration matrix, each capturing a dominant pattern of co-exposure across participants. The magnitude and direction of the loadings reflect how strongly and in what direction each chemical contributes to the underlying dimension. Component 1 is characterized by broadly negative loadings across chemical classes, with strong contributions from the metals like thallium and cesium. This pattern reflects a shared environmental or occupational exposure profile [18]. Component 2 is dominated by positive loadings for phthalate metabolites, these compounds frequently co-occur due to their widespread use in plastics, personal care products, and food contact materials. Component 3 reflects negative loadings for PFAS. The opposing directions of loadings across components further indicate that individuals with high exposure to one chemical group may have lower levels of others. In Component 3, for example, if lead has a positive loading and PFNA has a negative loading, individuals with high scores on this component are likely to have higher lead levels and lower PFNA levels. These latent co-exposure patterns highlight that individuals are exposed to chemical mixtures, not single substances, and that these mixtures follow identifiable structures based on shared sources and uses. And recognizing such patterns is essential for effective mixture-based risk assessment and public health action [7].

### 3.3. Associations Between Low-Rank Exposure Patterns and Hepatic Disease Risk

#### 3.3.1. Spearman Correlation Matrix of Urinary Biomarkers

**Figure 6:**
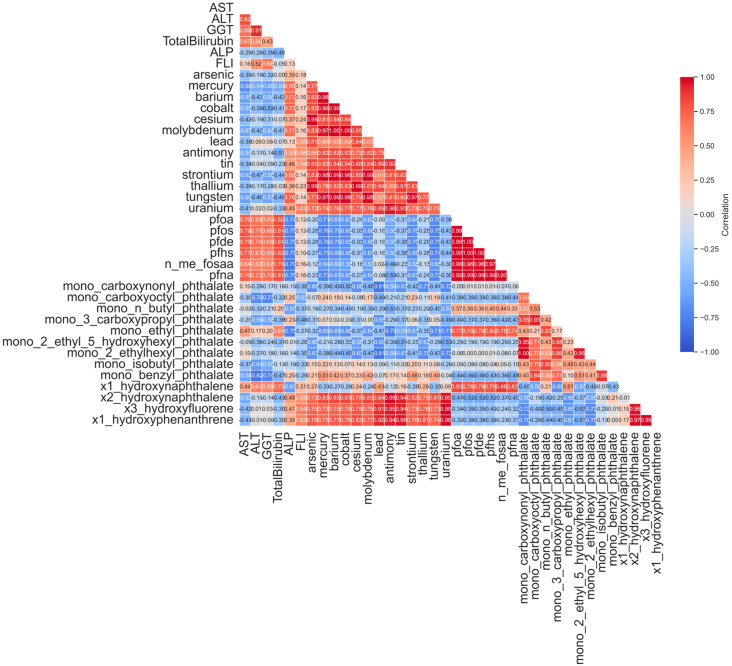
Rank-based correlations highlight co-exposure clusters among phthalates, PAH, PFAS, and metals.

Figure 6 shows the Spearman correlation matrix of all urinary biomarkers included in the BKMR model. This nonparametric measure captures monotonic relationships, making it ideal for skewed and left-censored environmental data [49]. Strong positive correlations are observed among phthalate metabolites (e.g., MEHHP, MBzP, MiBP), in line with shared sources like personal care products and PAH [50]. PFAS compounds (e.g., PFOS, PFNA) also cluster together, reflecting co-occurrence in drinking water and persistent industrial use [36]. Some metals show weaker or distinct correlations, suggesting different environmental or occupational origins [51].

#### 3.3.2. Posterior Inclusion Probabilities (PIPs) of Biomarkers for Hepatic Outcomes

**Figure 7:**
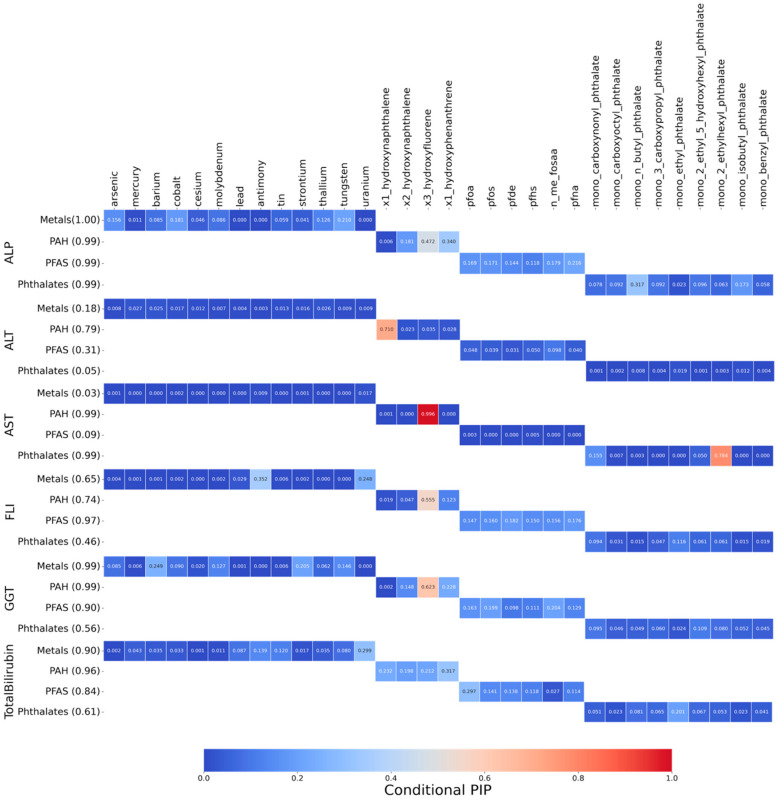
Posterior inclusion probabilities (PIPs) from BKMR models linking chemicals to liver biomarkers. Higher PIPs indicate greater relevance of a chemical to the outcome.

Figure 7 displays the PIPs derived from BKMR, indicating the likelihood that each chemical group contributes to variation in the six liver-related biomarkers. Higher PIPs suggest greater relevance in explaining the outcome, accounting for nonlinear and joint effects within the exposure mixture. Among chemical groups, phthalate metabolites showed low to moderate PIPs. PFAS compounds were also influential, particularly for AST and FLI, indicating their relevance to hepatic fat accumulation and systemic metabolic disturbance. In contrast, heavy metals contributed more variably across outcomes, but still showed meaningful inclusion probabilities for several markers, reinforcing concerns about metal-induced liver toxicity. Overall, the PIP matrix highlights that no single chemical class dominates across all outcomes. Instead, distinct liver biomarkers appear to be influenced by different chemical mixtures, reflecting divergent toxicological mechanisms. BKMR provides a framework to detect such group-level patterns, supporting more nuanced risk assessment strategies that move beyond single-compound analysis [21].

#### 3.3.3. Single-Variable Exposure Effects on Hepatic Outcomes from BKMR

**Figure 8:**
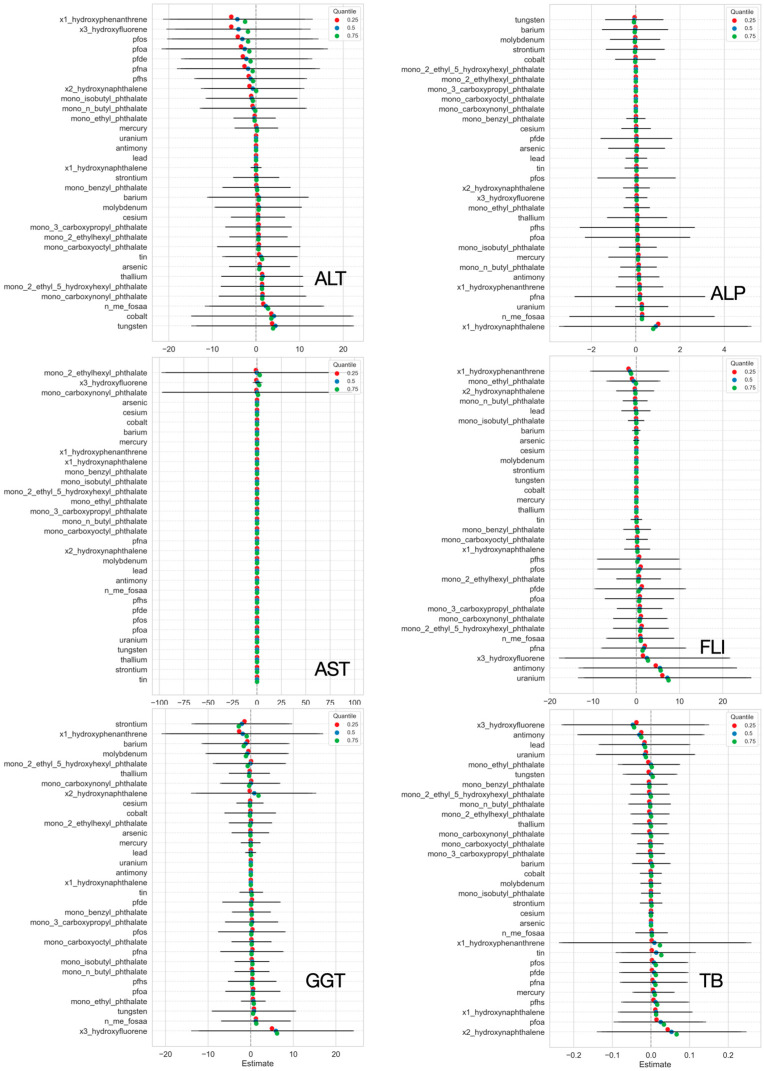
Estimated effects of individual chemicals on liver biomarkers, examining the change in response associated with a change in a single exposure from its 25th to 75th quantile while all other exposures are fixed at a specific quantile (25th, 50th, and 75th). Red represents 0.25 quantile, blue represents 0.50 quantile, and green represents 0.75 quantile. Black lines represent 95% credible intervals.

Figure 8 shows the estimated effects of individual urinary biomarkers on the six liver outcomes. Each plot shows how a chemical’s concentration shifts from the 25th to 75th percentile affects outcomes. Colored dots represent effect estimates at different percentiles, with 95% credible intervals shown as horizontal bars. Phthalates (e.g., Mono-(2-ethyl-5-hydroxyhexyl) are positively associated with ALP and FLI, reflecting potential liver injury, consistent with prior toxicological findings [52,53]. PFAS compounds (PFNA) show positive trends with ALT and FLI, suggesting links to fat accumulation and liver dysfunction [54]. PAHs such as 1-hydroxyphenanthrene and 3-hydroxyfluorene are also associated with elevated liver markers. Heavy metals show mixed effects, reflecting complex toxicokinetics [55]. These findings support the biological relevance of key environmental exposures and demonstrate BKMR’s utility in evaluating nonlinear effects in chemical mixtures [21].

#### 3.3.4. Individual Chemical Exposure Effects on Mixtures

**Figure 9:**
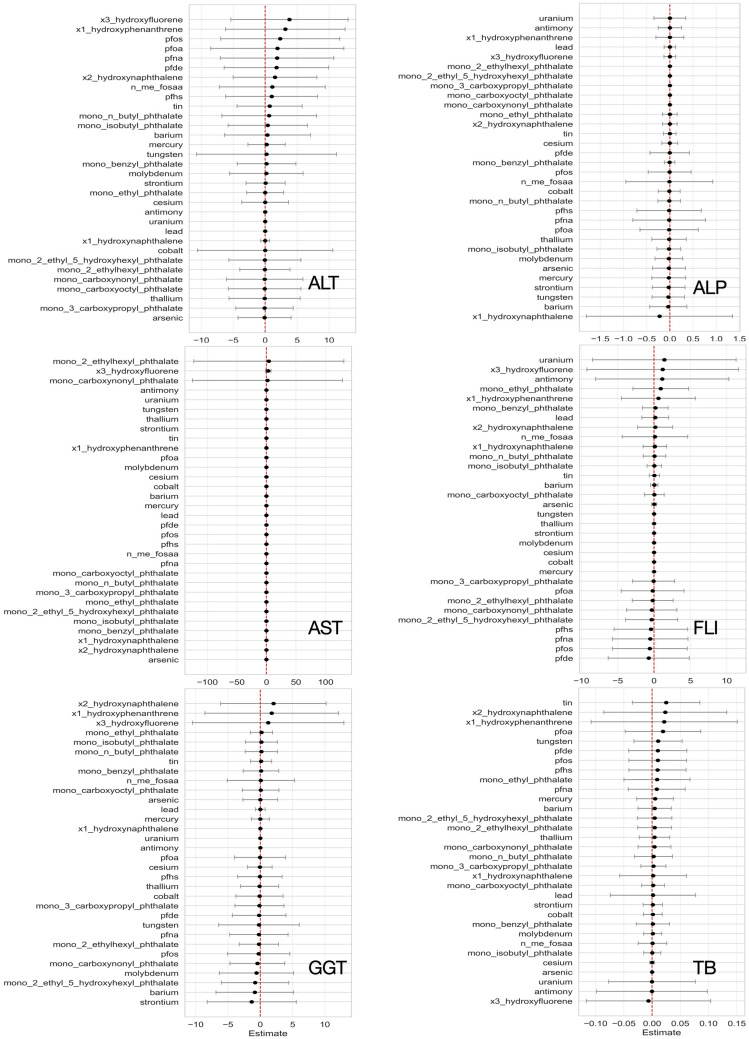
Individual exposure interaction analyses for liver markers assessing the effect of each environmental exposure (Metals, PFAS, Phthalates, and PAH) from its 25th to 75th percentile, when all others are fixed at the 25th percentile compared to the 75th percentile.

Figure 9 shows the estimated effects of individual urinary biomarkers on liver outcomes. Each plot illustrates how variations in a single chemical, while accounting for changes in all other exposures, influence liver biomarkers. PAH metabolites, particularly 3-hydroxyfluorene and 1-hydroxyphenanthrene, showed positive effects on ALP, FLI, and GGT. These results suggest a possible role in liver inflammation and stress, which has also been shown in earlier studies on PAHs and liver toxicity [56]. Some phthalates, such as mono-(2-ethylhexyl) and mono-ethyl, were also linked to AST and FLI. These effects are in line with previous research showing that phthalates can interfere with liver fat metabolism and increase the risk of liver damage [53]. PFAS compounds, including PFOS, PFNA, and PFOA, showed modest associations with ALP and total bilirubin, suggesting possible effects on liver function [54]. For trace metals like lead, mercury, and barium, the effects were more mixed, with both positive and negative associations across outcomes. This pattern reflects the complex ways these metals may act in the body and affect the liver depending on dose, timing, and co-exposures [55].

#### 3.3.5. Bivariate Exposure–Response Relationship

**Figure 10:**
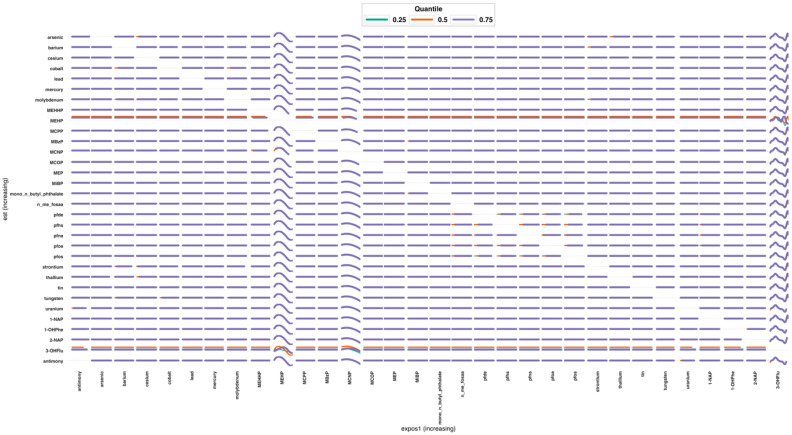
Bivariate exposure–response relationship for AST illustrates the joint association of increasing exposure of metals, PFAS, phthalate and PAH metabolites. Each panel shows the model-estimated change in the outcome (y-axis) as the column chemical increases (x-axis), while the row chemical is held at the 25th (green), 50th (orange), or 75th (purple) percentile.

Figure 10 is a matrix of bivariate exposure–response curves across the chemical mixture in relation to AST. Each row names the conditioning chemical and each column the varying chemical on the x-axis; all remaining chemicals are held at their medians. Within every panel, three curves show the predicted change in the outcome when the column chemical varies while the row chemical is fixed at the 25th (green), 50th (orange), or 75th (purple) percentile. Panels with nearly overlapping flat lines indicate little evidence of interaction or effect modification. Panels where the three lines are clearly separated or change shape (e.g., for several PAH metabolites and selected phthalates such as mono-2-ethyl across many columns) suggest stronger effects when the conditioning chemical is high, consistent with interaction-sensitive responses. The blank/neutral diagonal represents the univariate function (chemical with itself). Overall, most pairs show modest or flat relationships, while a subset involving PAHs and some phthalates exhibit stronger, higher-quantile (purple) responses, highlighting candidate co-exposures driving mixture effects. Results for ALT, ALP, GGT, FLI and total bilirubin are found in Supplementary Materials.

#### 3.3.6. Overall Effect of Chemical Mixture on Liver Health Outcomes

**Figure 11:**
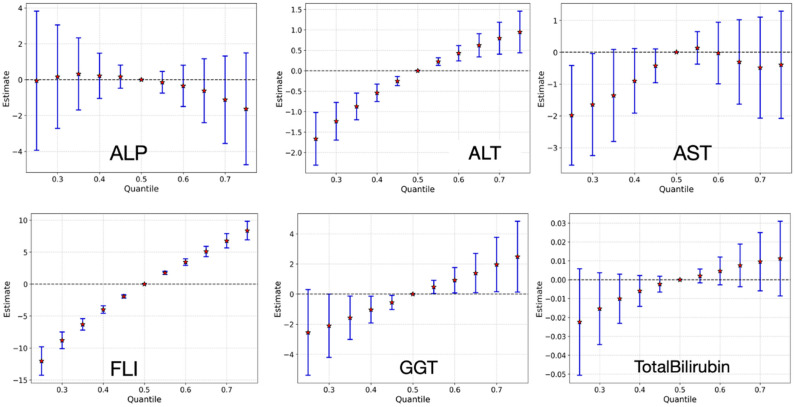
Estimated overall effect of increasing the combined chemical mixture from the 25th to the 75th percentile using BKMR. Each illustrates the dose–response relationship between total mixture exposure and a specific liver biomarker outcome.

Figure 11 presents the overall effects of increasing the combined exposure to the chemical mixture from the 25th to 75th percentile on six liver biomarkers, using estimates from BKMR. Notably, ALT and FLI exhibit clear upward trends, suggesting a positive association between higher mixture exposure and markers of liver injury, inflammation, and hepatic steatosis. These findings are consistent with emerging evidence that cumulative low-dose exposures can contribute to non-alcoholic fatty liver disease (NAFLD) and metabolic dysfunction [51,57]. GGT showed small, inconsistent associations across exposure percentiles, with wide uncertainty intervals and no clear trend. ALP and total bilirubin also displayed mixed patterns, which may reflect variability in how these biomarkers respond to environmental exposures or differences in their underlying biological mechanisms [58].

## 4. Discussion

This study applied PCP-LOD and BKMR to biomonitoring data from NHANES to characterize complex environmental mixtures and assess their associations with liver health. Environmental exposures rarely occur in isolation; instead, they co-occur in coherent, interpretable patterns driven by shared sources (e.g., drinking water, packaging, and personal care products) and common metabolic or environmental pathways, reinforcing the need for mixture-aware methods rather than single-pollutant analyses [1,3,21]. Many long-chain PFAS come from drinking water and diet and can build up along food webs (bioaccumulation/trophic transfer) [36]. Phthalate metabolites largely reflect contact with plastics and personal-care products, with diet and dermal absorption as key pathways [50]. PAHs arise from incomplete combustion (e.g., traffic, home heating) and from smoked or charred foods [56]. Several metals (e.g., thallium, lead, cadmium) are linked to geologic sources, industrial emissions, and contaminated media [34,35,38]. Framing our PCP-LOD groupings within these pathways clarifies why chemicals co-occur and why mixture-aware methods are needed.

Within the NHANES data, we focused on metals, PFAS, phthalate and PAH metabolites alongside clinically interpretable hepatic outcomes (AST, ALT, GGT, ALP, total bilirubin) and a composite steatosis indicator (FLI), which capture hepatocellular injury, cholestasis, and fatty liver risk [30].

PCP-LOD decomposed the censored, high-dimensional exposure matrix into a low-rank layer (population co-exposure profiles) and a sparse layer (rare, person-specific spikes). The low-rank layer showed coherent groupings across metals, PFAS, and phthalates within-class clusters like PFOS, PFNA, PFHS among PFAS and MEHHP, MBzP among phthalates [7,36,47,52]. The sparse layer flagged outlying elevations (e.g., mono-benzyl phthalate, PFOA), in line with episodic, occupational, or localized exposures, thereby separating broad patterns from unusual peaks. Methodologically, rather than replacing <LOD results with a fixed number or deleting them, PCP-LOD treats each non-detect as an inequality (value ≤ LOD) and estimates the exposure matrix under these constraints, decomposing it into a low-rank background and a sparse spike component. This uses all available information, reduces bias from substitution/deletion, and preserves the correlation patterns needed for mixture analysis [12,42]. The resulting low-rank factors provide interpretable exposure profiles (e.g., PFAS- or phthalate-dominated), while the sparse terms retain atypical events without distorting shared structure yielding higher-signal inputs for BKMR and improving detection of nonlinear and interactive effects. SVD applied to the PCP-LOD–denoised exposure matrix yielded a low-dimensional, interpretable map of co-exposure structure that accords with established source profiles and toxicokinetics. By performing the decomposition after censoring-aware denoising, the analysis reduced distortions from measurement error, non-detects, and missingness which are artifacts that commonly confound raw correlation matrices. In this latent space, metals such as arsenic, lead, cadmium, and barium and long-chain PFAS (e.g., PFOA, PFNA, PFHxS) form coherent clusters, while weak, non-robust pairings were attenuated, thereby sharpening biological interpretability.

Detection frequency patterns provided important context for interpreting mixture models. Many analytes were detected in >95% of participants, consistent with widespread population exposure and bioaccumulation, whereas others (e.g., PFHP, PFDO) were detected in <30% of participants, likely to reflect lower use, rapid elimination, or analytic sensitivity limits. To balance bias from left censoring, we set a priori inclusion threshold of ≥50% detection. This threshold reduces instability from ubiquitous non-detects while preserving coverage of chemicals relevant to public health. The sparse component isolated subject-specific exposure spikes that the low-rank structure cannot explain. Most notably, high deviations were concentrated among certain phthalates, suggesting episodic or product-specific surges, while PFOA, PFHS, and PFNA tended to present uniformly high, low or sparse signals. We operationalized a three-tier classification (Sparse, Low, High) that distinguishes values below LOD from quantifiable low-level exposure and from high events, which is crucial for realistic interpretation of censored biomonitoring matrices. These results show that PCP-LOD can resolve policy-relevant subgroups for intervention and environmental-justice evaluation, while conserving the underlying population pattern.

After characterizing co-exposure structure with PCP-LOD, we used BKMR to evaluate mixture–liver relationships because it can capture nonlinear exposure–response shapes, model interactions among many correlated pollutants, and operate effectively in high-dimensional data [21]. PIPs from the BKMR models (Figure 7) indicated that influence was distributed across classes: selected PAHs and phthalates showed higher inclusion, most notably for AST, ALT, GGT and FLI whereas metals exhibited modest, outcome-specific associations, with different metals aligning with different liver biomarkers, this concerns metal-related hepatotoxicity. Single-exposure (Figure 8), evaluated while holding the rest of the mixture at reference levels, supported these patterns: phthalate metabolites (e.g., mono-(2-ethyl-5-hydroxyhexyl)) were positively associated with ALP and FLI [52,53], PFAS (e.g., PFNA) tracked higher ALT and FLI [54], and PAH biomarkers (e.g., 1-hydroxyphenanthrene, 3-hydroxyfluorene) aligned with elevated ALT, GGT, total bilirubin, and FLI, while metals showed mixed directions [55]. Individual Chemical Exposure Interaction Effects on Mixtures (Figure 9) highlighted substantial heterogeneity across chemical classes. PAHs emerged as the most interaction-prone exposures, consistently showing positive associations with liver-related outcomes, including ALP, FLI, GGT, and total bilirubin, with effects that intensified when the background mixture was fixed at higher exposure levels. This pattern underscores their strong potential for synergistic effects, consistent with prior evidence of PAH-induced oxidative stress and hepatic injury in multi-pollutant contexts [56]. Phthalates, in contrast, showed little evidence of interaction, suggesting their associations with liver biomarkers were largely stable across mixture scenarios [52,53]. PFAS demonstrated modest interactions across most outcomes, but their interactive influence was particularly evident for ALP, where effects varied considerably by co-exposure level, while appearing more independent for total bilirubin [54]. Metals displayed minimal interaction effects, indicating largely additive contributions within the observed exposure range [55]. Collectively, these findings position PAHs and PFAS—particularly in relation to ALP—as critical interactive agents in mixture settings, while phthalates and metals appear to contribute more independently. Such distinctions are crucial for refining mixture risk assessment by differentiating exposures that drive synergistic responses from those that act primarily through additive mechanisms.

The overall mixture analysis (Figure 10) revealed that simultaneously shifting all exposures from the 25th to the 75th percentile produced outcome-specific patterns of response. The most pronounced effect was observed for FLI, which showed a clear upward trajectory, indicating that cumulative exposure across multiple pollutant classes contributes substantially to fatty liver risk. This finding is consistent with emerging evidence that low-to-moderate, chronic mixtures of environmental chemicals act synergistically to promote steatosis and metabolic dysregulation, even in the absence of high-level single-agent exposures. ALT also displayed an upward response, albeit more modest in magnitude, suggesting that transaminase elevations may be part of the subclinical signature of mixture-induced hepatic stress. In contrast, GGT exhibited only minor changes, which may reflect its greater sensitivity to alcohol intake and oxidative stress rather than to low-dose environmental mixtures per se. For ALP and total bilirubin, the overall response was mixed and less consistent, highlighting possible biomarker-specific differences in sensitivity to cumulative exposures, as well as potential compensatory or adaptive processes in hepatobiliary function. Together, these results reinforce the value of mixture-level modeling approaches, as they capture the aggregate effect of environmentally relevant co-exposures and demonstrate that the cumulative pollutant burden is most strongly expressed in markers of steatosis and metabolic dysfunction [51,57]. Overall, BKMR revealed class-specific yet outcome-dependent signals and emphasized that no single chemical class dominates across liver endpoints, supporting mixture-aware risk assessment that moves beyond single-compound inference.

Mechanistically, the observed associations are consistent with established pathways of hepatotoxicity across chemical classes [58]. Transition metals such as cadmium and lead can generate reactive oxygen species and disrupt mitochondrial electron transport, contributing to oxidative and inflammatory liver injury. PAHs similarly promote oxidative stress through cytochrome P450-mediated bioactivation and redox cycling [59]. Phthalates interfere with lipid and carbohydrate metabolism via peroxisome proliferator-activated receptor (PPAR) signaling, leading to altered lipid storage and steatosis [60]. Long-chain PFAS disrupts bile acid homeostasis and hepatic lipid transport, potentially driving cholestatic and steatotic responses [61]. Together, these mechanisms support the biological plausibility of the mixture effects observed in our study and suggest that overlapping oxidative, metabolic, and inflammatory pathways underlie the liver biomarker patterns identified.

Although the enzyme changes observed in this study are modest, their biological significance remains noteworthy. Population-level increases in ALT, AST, and GGT, even within clinically normal limits, have been associated with early hepatic stress, redox imbalance, and the initial stages of metabolic dysfunction and nonalcoholic fatty liver disease [62,63]. ALT and AST primarily reflect hepatocellular injury, whereas GGT is indicative of oxidative stress and alterations in biliary function. Likewise, slight elevations in the FLI may signal early lipid accumulation in hepatocytes before overt steatosis develops [64]. Therefore, the consistent changes observed in relation to chemical mixture exposure likely represent early hepatic disturbances with potential implications for long-term liver health.

This analysis has several notable strengths. First, the joint use of PCP-LOD and BKMR exploits complementary capabilities: PCP-LOD denoises censored, high-dimensional data to recover stable co-exposure structure, while BKMR flexibly estimates nonlinear and potentially synergistic mixture effects with full uncertainty quantification [21]. Second, decomposing the exposure matrix into low-rank latent patterns and a sparse anomaly component yields actionable insight for both broad prevention (source-like mixture profiles) and targeted mitigation (episodic, subgroup-specific spikes) [65]. Third, the large, population-based sample (n = 4367) improves precision and enhances the generalizability of the learned structures and their associations with liver outcomes. Fourth, censoring-aware cleaning reduced measurement noise and the influence of non-detects, making chemically plausible clusters more apparent and diminishing chance correlations; the a priori ≥50% detection threshold further limited non-biological patterns caused by measurement and imputation from heavy left-censoring. Finally, consistent signals from PCP-LOD/SVD and BKMR converged on the same chemical classes and patterns, strengthening confidence in the inferences.

Despite its contributions, the study has some limitations. Because NHANES is cross-sectional, exposures and outcomes are measured at the same time; therefore, we cannot determine whether exposure preceded the health change, and causal inference is limited. Because a single spot urine sample mainly reflects short-term exposure, pairing it with liver outcomes that evolve over months to years can misclassify long-term exposure and attenuate estimated associations. Our detection threshold can exclude emerging compounds with low prevalence, potentially underestimating their roles; conversely, inclusion of low-detect analytes without appropriate modeling of censoring would inflate noise. The overall pattern or structured co-exposures with multi-class contributions to liver markers proved robust across methods tailored to the data-quality constraints at hand. Despite these limitations, the study’s findings are biologically plausible and align with a growing body of literature linking environmental toxicants to hepatic risk factors. Recognizing these constraints tempers our conclusions and motivates future research for longitudinal designs and mechanistic investigations, to establish causality and probe interaction effects.

Future work should test the reproducibility of these latent exposure patterns across NHANES cycles and in longitudinal cohorts; quantify how much variance the low-rank factors explain across demographic strata; incorporate spatial information to relate components to industrial emissions, water systems, or product markets; and perform sensitivity analyses to kernels and priors in BKMR. Extending PCP-LOD to jointly model time-varying exposures and to propagate LOD uncertainty into health-effect estimates would further strengthen causal interpretation. The present findings already make a clear case: mixture-aware methods that respect censoring and non-linearity can recover meaningful exposure architectures and reveal clinically relevant links to liver injury and steatosis.

Future research should aim to integrate population-based mixture analyses with experimental validation to strengthen causal inference. Laboratory studies can be designed using the real-world chemical mixtures and concentration ranges identified from NHANES as empirically derived exposure “recipes.” These mixtures should be evaluated in controlled systems to elucidate key mechanistic pathways implicated in hepatic injury, including oxidative stress and inflammatory signaling commonly associated with PAHs [56], disruptions in lipid metabolism and peroxisomal function linked to phthalates [52,53], bile acid dysregulation and hepatic lipid accumulation reported for long-chain PFAS [54], and mitochondrial dysfunction and oxidative damage induced by select metals [55,57]. Integrating these experimental findings with population-level mixture models would enable bidirectional validation, providing mechanistic context for observed epidemiologic associations and refining the biological plausibility of mixture-driven liver effects.

## 5. Conclusions

This study demonstrates the utility of PCP-LOD and BKMR in uncovering latent co-exposure patterns and evaluating their associations with liver health. By addressing data censoring and exposure complexity, we identified key chemical mixtures like phthalates, PFAS, and metals, linked to hepatic biomarkers. These findings highlight the value of mixture-based methodologies in enhancing exposure risk assessment and guiding focused environmental health interventions.

These findings are useful for real-world monitoring. The PCP-LOD/SVD results give clear mixing patterns that can help target groups with higher PFAS or phthalate exposure. BKMR then shows how these mixtures relate to liver markers, including curved and combined (interaction) effects that single-chemical models miss. Together, they support mixture-aware risk assessment by flagging which chemical groups matter most and whether their effects add up or amplify each other. All analyses are fully scripted and can be rerun on other NHANES cycles or similar studies to check that the patterns hold and to guide public health action.

## Figures and Tables

**Table 1:** Baseline environmental exposure levels of 4367 participants from the 2013–2014 cycle of NHANES.

Chemical Class (Analytes Included)	n	Mean (SD)
Metals		
Arsenic (As, µg/L)	4367.0	16 (31)
Mercury (Hg, µg/L)	4367.0	1 (2)
Barium (Ba, µg/L)	4367.0	2 (2)
Cobalt (Co, µg/L)	4367.0	1 (2)
Cesium (Cs, µg/L)	4367.0	5 (2)
Molybdenum (Mo, µg/L)	4367.0	52 (41)
Lead (Pb, µg/L)	4367.0	0.5 (1)
Antimony (Sb, µg/L)	4367.0	0.07 (0.11)
Tin (Sn, µg/L)	4367.0	1.33 (4)
Strontium (Sr, µg/L)	4367.0	122.33 (152.25)
Thallium (TI, µg/L)	4367.0	0.18 (0.10)
Tungsten (W, µg/L)	4367.0	0.12 (0.20)
Uranium (U, µg/L)	4367.0	0.01 (0.03)
PFAS		
Perfluorooctanoic acid (PFOA) (ng/mL)	4367.0	2.30 (2.19)
Perfluorooctanesulfonic acid (PFOS) (ng/mL)	4367.0	8.15 (24.00)
Perfluorodecanoic acid (PFDE) (ng/mL)	4367.0	0.30 (1.00)
Perfluorohexanesulfonic acid (PFHS) (ng/mL)	4367.0	1.95 (1.70)
Perfluorononanoic acid (PFNA) (ng/mL)	4367.0	0.87 (0.63)
N-Methyl perfluorooctane sulfonamidoacetic acid (NMeFOSAA)	4367.0	0.18 (0.22)
Perfluoroheptanoic acid (PFHP)	4367.0	0.08 (0.04)
Perfluoroundecanoic acid (PFUA)	4367.0	0.22 (1.58)
Perfluorododecanoic acid (PFDO)	4367.0	0.09 (0.13)
Phthalates		
Mono-ethyl phthalate (MEP) (ng/mL)	4367.0	204.46 (874.78)
Mono-n-butyl phthalate (MnBP) (ng/mL)	4367.0	17.45 (20.07)
Mono-isobutyl phthalate (MiBP) (ng/mL)	4367.0	14.06 (17.01)
Mono-benzyl phthalate (MBzP) (ng/mL)	4367.0	9.93 (14.49)
Mono-(2-ethyl-5-carboxypentyl) phthalate (ng/mL)	4367.0	0.11 (0.23)
Mono-(2-ethyl-5-hydroxyhexyl) phthalate (ng/mL)	4367.0	12 (22)
Mono-(2-ethyl-5-hydroxynonyl) phthalate (ng/mL)	4367.0	1 (3)
Mono-(3-carboxypropyl) phthalate (MCPP) (ng/mL)	4367.0	5 (12)
Mono (carboxynonyl) phthalate (ng/mL)	4367.0	6 (21)
Mono (carboxyoctyl) phthalate (ng/mL)	4367.0	53 (85)
PAH		
1-Hydroxynaphthalene (ng/L)	4367.0	29,726 (581,208)
2-Hydroxynaphthalene (ng/L)	4367.0	9109 (11,183)
2-Hydroxyfluorene (ng/L)	4367.0	425 (605)
3-Hydroxyfluorene (ng/L)	4367.0	241 (394)
1-Hydroxyphenanthrene (ng/L)	4367.0	149 (180)
1-Hydroxyphenol (ng/L)	4367.0	197 (238)

## Data Availability

The NHANES dataset is publicly available online, accessible at https://wwwn.cdc.gov/nchs/nhanes/continuousnhanes/overview.aspx?BeginYear=2013 (accessed on 12 September 2025).

## Supplementary Materials

The following supporting information can be downloaded at https://www.mdpi.com/article/10.3390/jox15060178/s1, Figure S1: Bivariate exposure–response relationship for ALP illustrating the joint association of increasing exposure to metals, PFAS, phthalate, and PAH metabolites; Figure S2: Bivariate exposure–response relationship for ALT illustrating the joint association of increasing exposure to metals, PFAS, phthalate, and PAH metabolites; Figure S3: Bivariate exposure–response relationship for FLI illustrating the joint association of increasing exposure to metals, PFAS, phthalate, and PAH metabolites; Figure S4: Bivariate exposure–response relationship for GGT illustrating the joint association of increasing exposure to metals, PFAS, phthalate, and PAH metabolites; Figure S5: Bivariate exposure–response relationship for Total Bilirubin illustrating the joint association of increasing exposure to metals, PFAS, phthalate, and PAH metabolites.

## Abbreviations

The following abbreviations are used in this work:

Study design, datasets, and statistics	
NHANES	National Health and Nutrition Examination Survey
NCHS	National Center for Health Statistics
PCP	Principal Component Pursuit
PCP-LOD	Principal Component Pursuit with Limits of Detection
BKMR	Bayesian Kernel Machine Regression
PIP/PIPs	Posterior Inclusion Probability/Probabilities
PCA	Principal Component Analysis
FA	Factor Analysis
SVD	Singular Value Decomposition
LOD	Limit of Detection
**Chemical classes and representative analytes**	
PFAS	Per- and Polyfluoroalkyl Substances
PAH/PAHs	Polycyclic Aromatic Hydrocarbon(s)
PFOA	Perfluorooctanoic Acid
PFOS	Perfluorooctanesulfonic Acid
PFDE	Perfluorodecanoic Acid
PFNA	Perfluorononanoic Acid
PFHP	Perfluoroheptanoic Acid
PFUA	Perfluoroundecanoic Acid
PFDO	Perfluorododecanoic Acid
PFHS	Perfluorohexanesulfonic Acid (often written PFHxS in the literature)
PFHxA	Perfluorohexanoic Acid
PFBA	Perfluorobutanoic Acid
NMeFOSAA	N-Methyl Perfluorooctane Sulfonamidoacetic Acid
MEP	Mono-ethyl Phthalate
MnBP	Mono-n-butyl Phthalate
MiBP	Mono-isobutyl Phthalate
MBzP	Mono-benzyl Phthalate
MEHHP	Mono-(2-ethyl-5-hydroxyhexyl) Phthalate
MCPP	Mono-(3-carboxypropyl) Phthalate
(Other phthalate metabolite names expanded in Table 1 remain as written in the manuscript.)	
Liver outcomes and clinical indices	
AST	Aspartate Aminotransferase
ALT	Alanine Aminotransferase
GGT	Gamma-Glutamyl Transferase
ALP	Alkaline Phosphatase
FLI	Fatty Liver Index
MASLD	Metabolic Dysfunction-Associated Steatotic Liver Disease
NAFLD	Non-Alcoholic Fatty Liver Disease
TG	Triglycerides
BMI	Body Mass Index
Laboratory methods/instruments	
ICP-MS	Inductively Coupled Plasma–Mass Spectrometry
HPLC-MS/MS	High-Performance Liquid Chromatography–Tandem Mass Spectrometry
HPLC-ESI-MS/MS	High-Performance Liquid Chromatography–Electrospray Ionization–Tandem Mass Spectrometry
API (5500)	Atmospheric Pressure Ionization mass spectrometer platform (AB Sciex)
