# Supplementary material for: Uncovering Exposure Patterns of Metals, PFAS, Phthalates, and PAHs and Their Combined Effect on Liver Injury Markers
Source: J Xenobiot. 2025 Nov 1;15(6):178. doi: 10.3390/jox15060178 (PMC12641841; doi:10.3390/jox15060178)
Supplement: Supplementary file 1 [file jox-15-00178-s001.zip › jox-3901057-supplementary.pdf]

Supplementary Materials: Uncovering Exposure Patterns of Metals, PFAS, Phthalates, and PAHs and Their Combined Effect on Liver Injury Markers

Doreen Jehu-Appiah and Emmanuel Obeng-Gyasi

The bivariate exposure-response function results for ALP, ALT, FLI, GGT and Total Bilirubin are shown in supplementary figures 1-5 below.

ALP

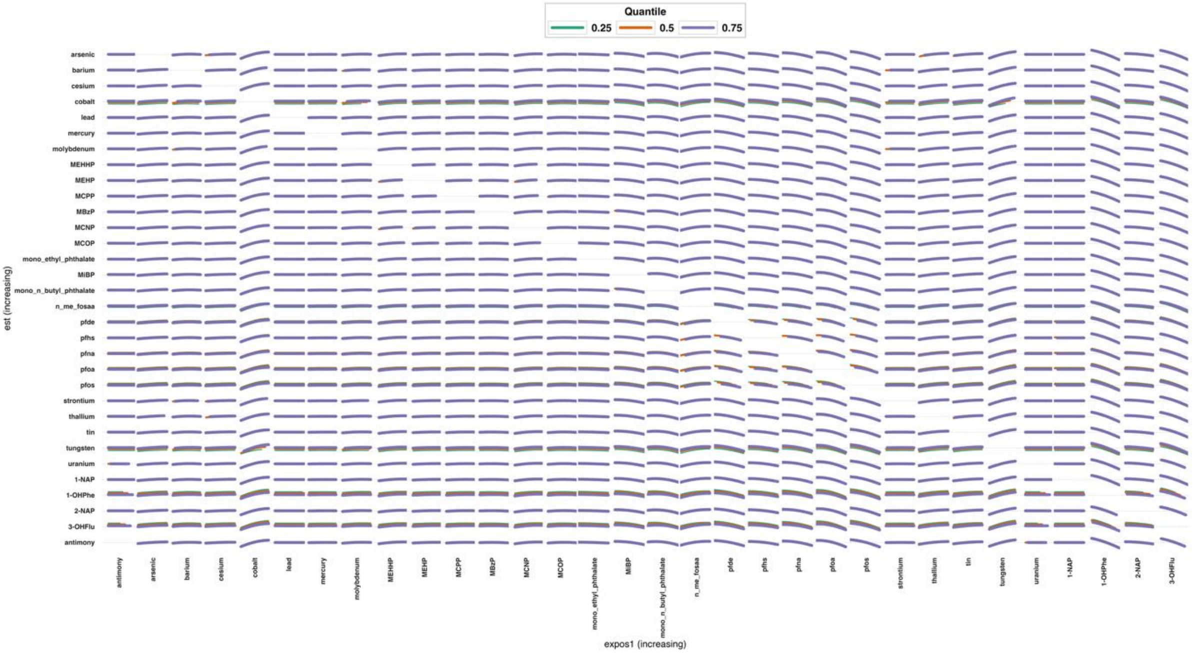

Figure S1. Bivariate exposure–response relationship for ALP illustrates the joint association of increasing exposure of metals, PFAS, phthalate and PAH metabolites. Each panel shows the model-estimated change in the outcome (y-axis) as the column chemical increases (x-axis), while the row chemical is held at the 25th (green), 50th (orange), or 75th (purple) percentile.

ALT

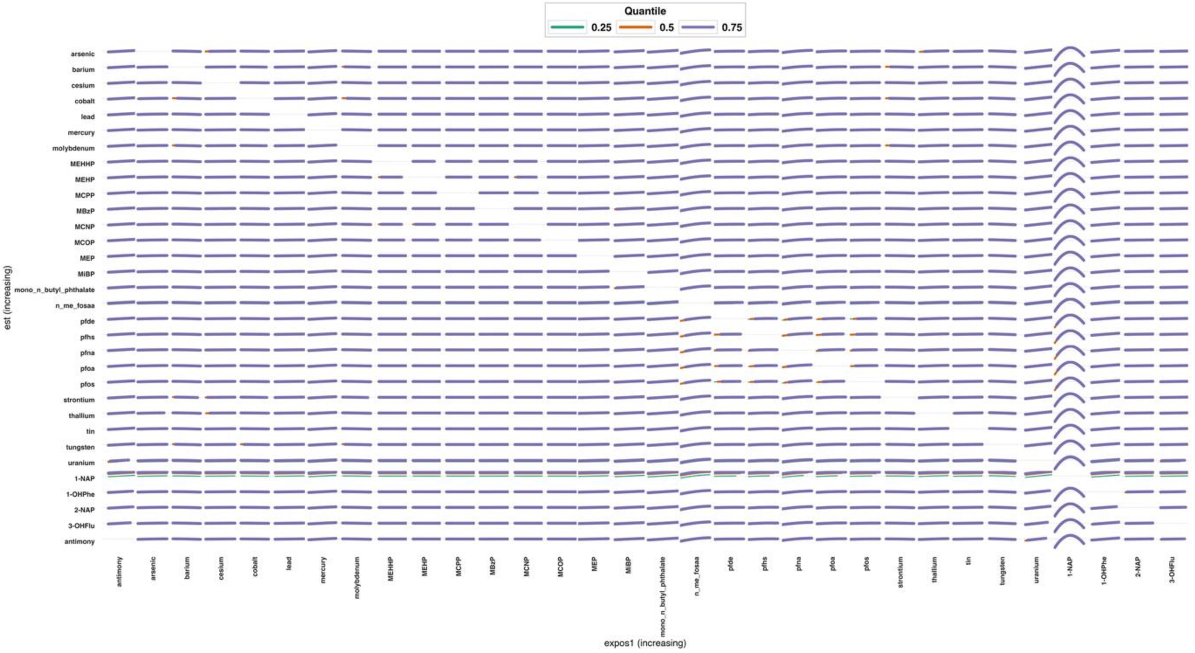

Figure S2. Bivariate exposure–response relationship for ALT illustrates the joint association of increasing exposure of metals, PFAS, phthalate and PAH metabolites. Each panel shows the model-estimated change in the outcome (y-axis) as the column chemical increases (x-axis), while the row chemical is held at the 25th (green), 50th (orange), or 75th (purple) percentile.

FLI

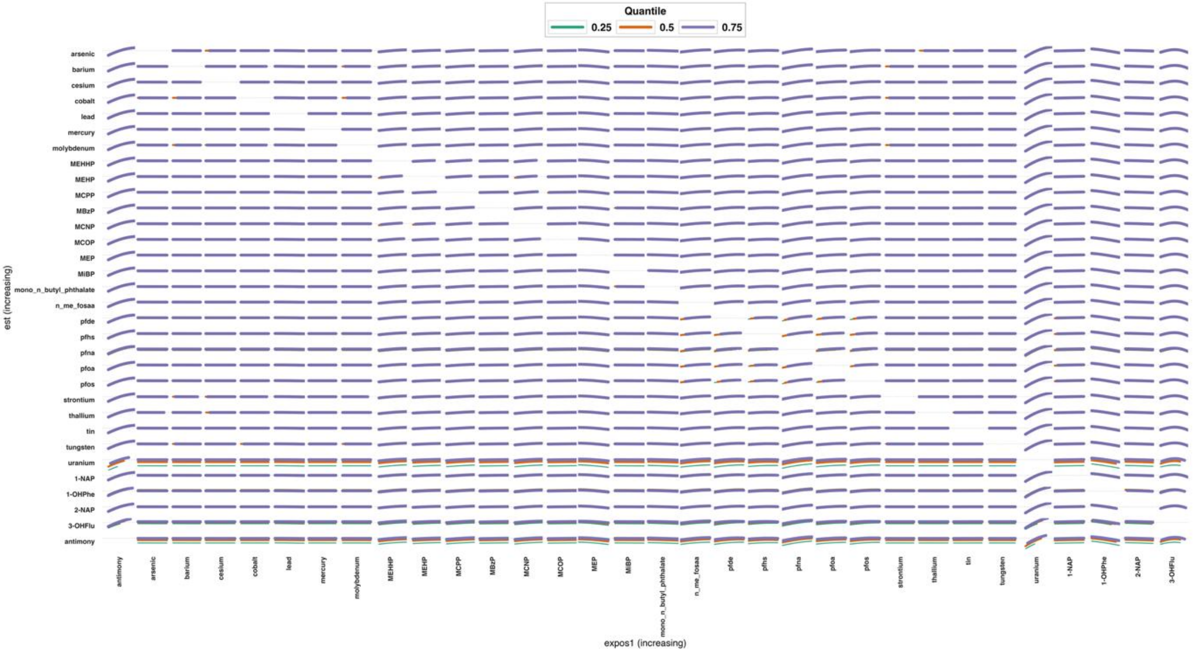

Figure S3. Bivariate exposure–response relationship for FLI illustrates the joint association of increasing exposure of metals, PFAS, phthalate and PAH metabolites. Each panel shows the model-estimated change in the outcome (y-axis) as the column chemical increases (x-axis), while the row chemical is held at the 25th (green), 50th (orange), or 75th (purple) percentile.

GGT

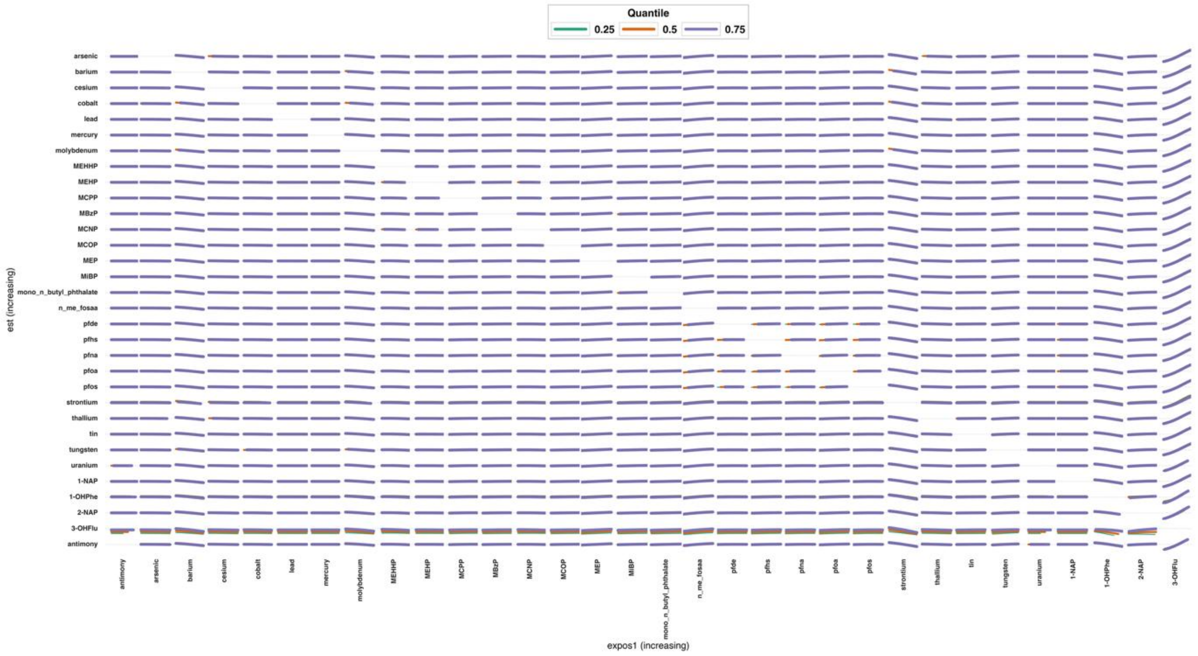

Figure S4. Bivariate exposure–response relationship for GGT illustrates the joint association of increasing exposure of metals, PFAS, phthalate and PAH metabolites. Each panel shows the model-estimated change in the outcome (y-axis) as the column chemical increases (x-axis), while the row chemical is held at the 25th (green), 50th (orange), or 75th (purple) percentile.

Total Bilirubin (TB)

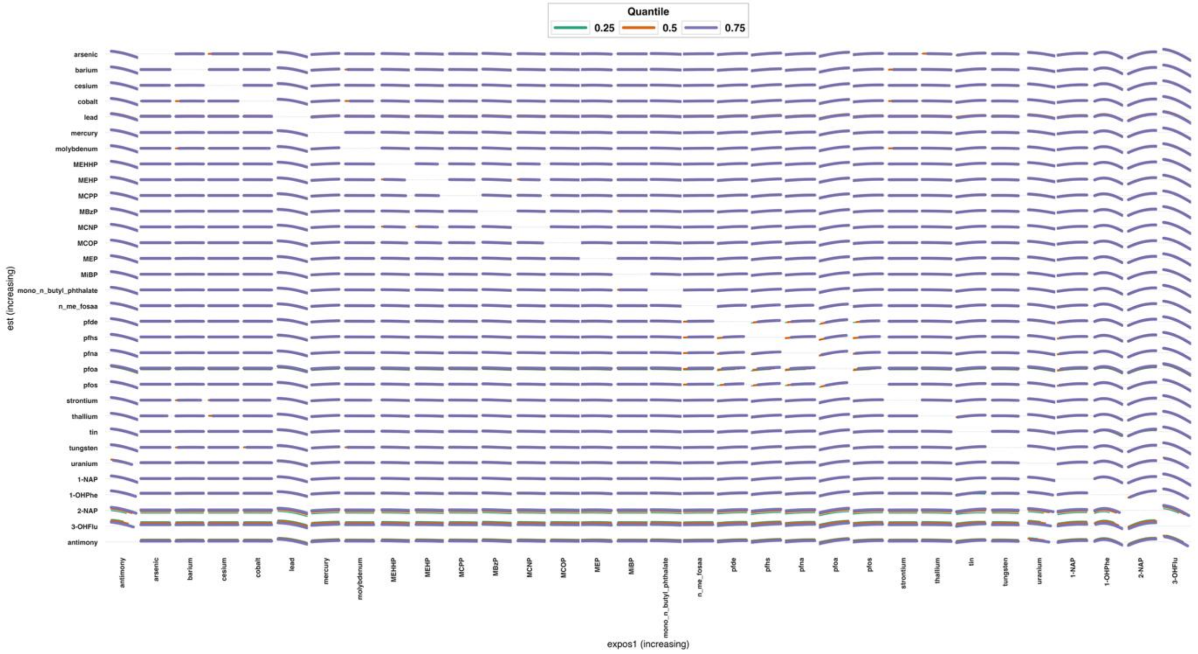

Figure S5. Bivariate exposure–response relationship for Total Bilirubin illustrates the joint association of increasing exposure of metals, PFAS, phthalate and PAH metabolites. Each panel shows the model-estimated change in the outcome (y-axis) as the column chemical increases (x-axis), while the row chemical is held at the 25th (green), 50th (orange), or 75th (purple) percentile.
